# Risk factors for ESBL-producing *Escherichia coli* on pig farms: A longitudinal study in the context of reduced use of antimicrobials

**DOI:** 10.1371/journal.pone.0174094

**Published:** 2017-03-21

**Authors:** Wietske Dohmen, Alejandro Dorado-García, Marc J. M. Bonten, Jaap A. Wagenaar, Dik Mevius, Dick J. J. Heederik

**Affiliations:** 1 Department of Environmental Epidemiology, Institute for Risk Assessment Sciences, Utrecht University, Utrecht, the Netherlands; 2 Department of Infectious Diseases and Immunology, Faculty of Veterinary Medicine, Utrecht University, Utrecht, the Netherlands; 3 Department of Medical Microbiology, University Medical Centre, Utrecht, the Netherlands; 4 Julius Centre for Health Sciences and Primary Care, University Medical Centre, Utrecht, the Netherlands; 5 Central Veterinary Institute of Wageningen University and Research Centre, Lelystad, the Netherlands; Ross University School of Veterinary Medicine, SAINT KITTS AND NEVIS

## Abstract

The presence of extended-spectrum beta-lactamase-producing *Escherichia coli* (ESBL-*E*. *coli*) in food animals is a public health concern. This study aimed to determine prevalence of ESBL-*E*. *coli* on pig farms and to assess the effect of reducing veterinary antimicrobial use (AMU) and farm management practices on ESBL-*E*. *coli* occurrence on pig farms. During 2011–2013, 36 Dutch conventional pig farms participated in a longitudinal study (4 sampling times in 18 months). Rectal swabs were taken from 60 pigs per farm and pooled per 6 pigs within the same age category. Presence of ESBL-*E*. *coli* was determined by selective plating and ESBL genes were characterized by microarray, PCR and gene sequencing. An extensive questionnaire on farm characteristics and AMU as Defined Daily Dosages per Animal Year (DDDA/Y) was available for the 6-month periods before each sampling moment. Associations between the presence of ESBL-*E*. *coli*-positive pigs and farm management practices were modelled with logistic regression. The number of farms with ESBL-*E*. *coli* carrying pigs decreased from 16 to 10 and the prevalence of ESBL-*E*. *coli*-positive pooled pig samples halved from 27% to 13%. Overall, the most detected ESBL genes were *bla*_CTX-M-1_, *bla*_TEM-52_ and *bla*_CTX-M-14_. The presence of ESBL-*E*. *coli* carrying pigs was not related to total AMU, but it was strongly determined by the presence or absence of cephalosporin use at the farm (OR = 46.4, p = 0.006). Other farm management factors, related with improved biosecurity, were also plausibly related to lower probabilities for ESBL-*E*. *coli*-positive farms (e.g. presence of a hygiene lock, pest control delivered by a professional). In conclusion, ESBL-*E*. *coli* prevalence decreased in pigs during 2011 and 2013 in the Netherlands. On pig farms, the use of cephalosporins was associated with the presence of ESBL-*E*. *coli* carrying pigs.

## Introduction

A variety of ESBLs have been identified in Enterobacteriaceae derived from food-producing animals worldwide [1]. High antimicrobial use (AMU) and inappropriate use of cephalosporins in livestock production are considered to be associated with the emergence and high prevalence of ESBL-producing *Escherichia coli* (ESBL-*E*. *coli*) in animals [2]. Transmission of ESBL genes from animals to humans can occur through food or direct contact [3,4]. Infections with ESBL-*E*. *coli* are a major global public health concern [5].

Several European studies reported high proportions of pig farms where ESBLs were present. In Spain, ESBL-*E*. *coli* were detected in faecal samples collected from stable floors of 8 out of 10 farms [6]. Two German studies found ESBL-*E*. *coli* in faecal samples collected from pigs on 15 out of 17 and 26 out of 35 farms respectively [7,8]. In a Danish study ESBLs were detected in pigs on 15 out of 19 pig farms with high consumption of cephalosporins versus 4 out of 20 pig farms with no cephalosporin use [9].

A reduction in AMU, more specifically cephalosporins, is suggested to decrease ESBL-*E*. *coli* on pig farms [2,9,10]. Because of demands regarding reduction in AMU in livestock production by the Dutch government, the total consumption of antimicrobials by animals dropped drastically in the Netherlands since 2011 [11–14]. Moreover, in 2011 the Dutch pig farm sector introduced a private initiative to stop the use of all cephalosporins (Dutch pig farms only used 3^rd^ generation cephalosporins). Additionally, from January 2013, veterinarians were legally required to limit the use of 3^rd^/4^th^ generation cephalosporins and fluoroquinolones to infections confirmed by bacteriological culture and susceptibility tests. As a consequence, at the vast majority of pig farms cephalosporins are not used anymore since 2011 [12–15]. Although not studied until now, other management practices besides reduction in AMU might have an effect on the presence of ESBL-*E*. *coli* on pig farms as well.

The objectives of this longitudinal study were to determine the prevalence of ESBL-*E*. *coli* on pig farms and to assess the effect of AMU reduction and farm management practices on the presence of ESBL-*E*. *coli* on pig farms.

## Materials and methods

### Study design

The design of the study has been described elsewhere [4,16]. Briefly, 36 multiplier pig farms (sows and piglets present), with or without finishing pigs, completed the study. Production types were classified in *farrowing* and *farrow-to-finish farms*. Farrowing farms did not produce fatteners and they delivered piglets to finishing farms (with the exception of one farm delivering gilts for farrowing). Farrow-to-finishing farms integrated farrowing and finishing pig production and delivered fattening pigs to the abattoir. Additionally, a farm was defined as *open* when receiving external supply of gilts for at least once a year from at least one supplier, and as *closed* when there was no external supply of gilts.

Farms and veterinarians were visited at the start of the study by the researcher between March 2011 and September 2011. At four sampling moments over a period of 18 months (6-month intervals), rectal samples from 60 pigs were collected by the farm veterinarian, using sterile cotton-wool swabs (Cultiplast^®^) and sent refrigerated to the laboratory by courier. All animal age groups present were sampled: sows, gilts, suckling piglets, weaning piglets and finishing pigs. Rectal swabs were combined in 10 pools (2 per age category) of 6 pigs. When no finishing pigs were present, weaning piglets were sampled instead. Each pool consisted of an age group in the same pen. Approval from an animal ethics committee was not required. The collection of rectal swabs from animals was in compliance with the Dutch law for animal welfare and did not fall under the Dutch Experiments on Animals Act (1996) or Directive 2010/63/EU. At the first sampling moment (baseline measurements), a questionnaire was completed during a walk through survey by the farm veterinarian to identify which management aspects could be improved to reduce antimicrobial resistant bacteria. The questionnaire (S1 Table) contained items on farm characteristics, biosecurity, animal management and hygiene practices and can also be found elsewhere [16]. A tailor-made intervention protocol was developed by the veterinarian and the farmer. Interventions were focused on improving personnel and farm hygiene, changing animal contact structures, and reducing AMU (in a background of decreasing AMU nationwide due to government demands). At each sampling moment the farm questionnaire was filled out again to monitor changes in farm practices.

### Laboratory analysis

All samples were analysed as described previously, namely pooled swabs were analysed for the presence of ESBL-producing Enterobacteriaceae by selective plating [4]. Samples were suspended in 10 ml peptone water and incubated overnight at 37°C. For screening of ESBL-producing Enterobacteriaceae, suspensions were cultured on selective agar plates (*Brilliance*^™^ ESBL Agar, Oxoid^®^) and incubated overnight at 37°C aerobically. When no growth was seen, plates were incubated another night at 37°C. Morphologically different colonies suspected of ESBL production were cultured individually on blood agar plates (Oxoid^®^) and incubated overnight at 37°C. In case of morphological uncertainty an oxidase test was performed before culturing. Bacterial species identification of the isolates was performed by MALDI/TOF (Bruker^®^). For phenotypical confirmation of the presence of ESBL-producing Enterobacteriaceae, a 0.5 McFarland suspension was inoculated on a Mueller Hinton agar and a combination disc test (ROSCO^®^) including cefotaxime, cefotaxime+clavulanate, ceftazidime, ceftazidime+clavulanate, cefepime, and cefepime+clavulanate (*Neo-Sensitabs*^™^) was used (according to the guidelines of the manufacturer (http://www.rosco.dk)). Isolates were stored at -80°C.

In earlier cross-sectional research on the first sampling moment, most Enterobacteriaceae other than *E*. *coli* with ESBL phenotype did not harbor ESBL genes [4]. Therefore, only phenotypically confirmed ESBL-*E*.*coli* were selected for further molecular analysis in the remaining sampling moments. In all ESBL-*E*. *coli* the presence and characteristics of the of ESBL genes was identified by PCR and sequence analysis. DNA was isolated using UltraClean^®^ Microbial DNA Isolation Kit (MO BIO Laboratories, Inc.) or DNeasy 96 Blood & Tissue Kit (Qiagen). Real-Time PCR (SybrGreen, Life Technologies), conventional PCR (BioMix Red, Bioline) and a *bla*_CTX-M_ group 1 specific PCR [17] was used to detect presence of the ESBL gene groups *bla*_CTX-M-1_, *bla*_TEM_, *bla*_SHV_ and *bla*_CMY-2_. Isolates with a negative PCR result were analysed using ESBL microarray (Check-MDR CT101, Checkpoints, Wageningen) to detect other ESBL gene groups. DNA from PCR or ESBL microarray positive isolates was sequenced with group-specific primers to determine the exact gene type. DNA sequences were interpreted with Basic Local Alignment Search Tool (National Center for Biotechnology Information).

### Data on antimicrobial use

The Defined Daily Dosage Animal per Year (DDDA/Y) is a standard weighted measure which can be interpreted as the number of days of antibiotic use per year for an average animal or animal place. A more detailed description on the calculation of DDDA/Y is described in the Netherlands Veterinary Medicines Authority report and by Bos et al.[11,14]. Data on AMU for the farms in this study have been described elsewhere [16]. In short, all antimicrobial prescriptions made to each farm were retrieved from the sector quality system national databases. AMU was expressed DDDA/Y per farm for the four periods preceding each sampling moment. Data was also available on wether the treatment was given individually or as group treatment. Since the use of cephalosporin was incidental, a new variable was created classifying farms with or without any cephalosporin use during the study period.

### Data analysis

Statistical analyses were performed using SAS version 9.2 (SAS Institute Inc., Cary, NC, USA). Farms were classified as ESBL-positive if an ESBL gene was detected in at least one *E*. *coli* isolate from a pooled pig sample. One farm in the last sampling moment was classified ESBL-positive based on one phenotypically confirmed ESBL-*E*. *coli*, since the isolate was lost before molecular analysis. Changes in presence of ESBL-producing *E*. *coli* on a farm and AMU over time were explored using simple descriptive statistics. DDDA/Y was log_2_ transformed because of its right-skewed distribution. Univariate longitudinal analysis was performed with AMU and farm questionnaire variables which had less then 10% missing values and more than 10% of farms present in each category. A total of 134 variables in the farm questionnaire (S1 Table) were selected together with AMU and cephalosporin use. The associations between presence of ESBL-*E*. *coli* on the farm and AMU, cephalosporin use and other farm variables was calculated with generalized linear mixed models (PROC GLIMIX; SAS Institute, Inc.) with random intercept for farms, taking into account the dependency of the data in a repeated measurements design. The univariate analysis was done for all the farms and for open and close farms separately; only associations from all farms with p≤0.2 from the questionnaire were presented. Pairwise Spearman correlations in questionnaire variables from the univariate analysis with p≤0.1 together with AMU and sampling time were checked to construct a full model. The final model was the result of a backward elimination from the full model, except for sampling time and AMU in DDDA/Y which were forced in the model during all steps because of special interest a priori. The final model retained variables significant at p≤0.05, again except for sampling moment and AMU. Model assumptions were checked with diagnostic plots. Variables from the full model at farm level were used to make a model at pooled pig sample level (i.e. modelling probabilities for a pooled pig sample to test ESBL-positive); this way we adjusted for age group of the animals. The latter model accounted for clustering at the farm level.

## Results

### Presence of ESBL-*E*.*coli* on pig farms

A description of the 36 farms is presented in Table 1. The number of farms where ESBL-*E*. *coli* carrying pigs were present decreased significantly from 16 farms at the beginning of the study (month 0) to 10 positive farms in the last sampling moment (month 18). Nineteen farms were negative for ESBL-*E*. *coli* during the whole study (8 farrow-to-finish closed, 5 farrowing open, 4 farrow-to-finish open and 2 farrowing closed). Eight farms were ESBL-*E*. *coli*-positive in all sampling moments (6 farrow-to-finish open, 1 farrow-to-finish closed and 1 open farrowing farm). Seven farms became negative during the study (3 farrowing open, 1 farrow-to-finish open, 2 farrow-to-finish closed and 1 farrowing close). One farrow-to-finish open farm became ESBL-*E*. *coli*-positive during the course of the study. The median number of ESBL-*E*. *coli*-positive pooled samples among the 10 collected per farm and per sampling time was 0 (IQR = 0–3, percentile 95^th^ = 8).

**Table 1:** Farm characteristics.

Type of farm	No. of farms	Median no. (interquartile range)	
		Sows	Fatteners
**All farms**	36	350 (270–550)	773 (0–1950)
**Open farms**^a^	22	337 (300–500)	500 (0–1300)
Farrowing^b^	9	533 (350–800)	-
Farrow-to finish	13	314 (242–380)	1100 (600–2010)
**Closed farms**^a^	14	407 (232–698)	1400 (450–2725)
Farrowing^b^	3	439 (239–905)	-
Farrow-to finish	11	367 (200–673)	1892 (1025–2950)

^a^ Farms were defined as open when they received external supplies of gilts ≥1 time per year from at least 1 supplier and as closed when they received no external supply of gilts.

^b^ No fattening pigs present.

**Fig 1:**
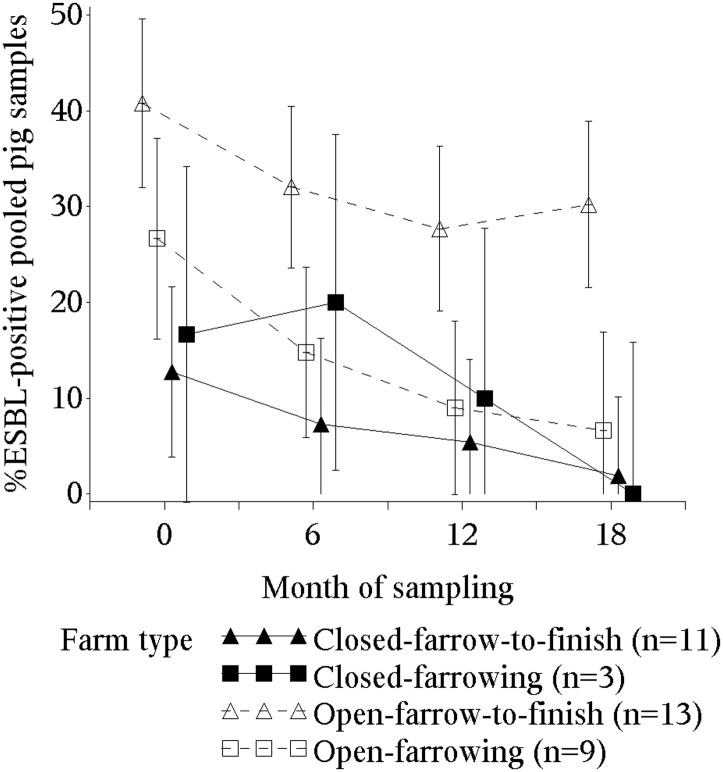
Prevalence of ESBL-*E*. *coli*-positive pooled samples from pigs per farm type. Error bars indicate 95% CIs.

A pronounced and statistically significant drop in prevalence of ESBL-*E*. *coli* was observed over the study period. The proportion of ESBL-*E*. *coli*-positive pooled pig samples in all farms halved from 27% at the first to 13% at the last sampling moment. Farrow-to-finish open farms showed a clear higher prevalence as compared to other farm types (Fig 1).

ESBL-*E*. *coli* carriage significantly differed between the sampled age groups. Overall ESBL-*E*. *coli* prevalence in pooled pig samples ranged from 11.7% in (rearing) gilts to 24.2% in sucking piglets (Table 2). The prevalence decreased parallel across all age groups (results not shown). Mostly *bla*_CTX-M-1_ genes were detected in pig isolates. Other ESBL genes found were *bla*_TEM-52_, *bla*_CTX-M-14_, *bla*_CTX-M-15_, *bla*_CTX-M-2_ and *bla*_CTX-M-32_ (Table 3).

**Table 2:** Prevalence of ESBL-*E*. *coli* in pooled pig samples within different age groups.

Age group	Pooled pig samples (n)	Pooled pig samples with presence of ESBL-*E*. *coli* (n and %)
**Sows**	283	60 (21.2)
**(Rearing) gilts**	281	33 (11.7)
**Suckling piglets**^a^	285	69 (24.2)
**Weaned piglets**	318	66 (17.2)
**Finishing pigs**	183	31 (16.9)

^a^ Suckling piglets = pooled pig sample contained rectal swabs from one mother sow and five of her suckling piglets.

**Table 3:** Distribution of ESBL genes in isolates from pooled pig samples.

Sampling time	*bla*_CTX-M-1_	*bla*_TEM-52_	*bla*_CTX-M-14_	*bla*_CTX-M-15_	Other^a^	Total
**0 mo**	81^b^	24	18	11	5	139
**6 mo**	57	15	3	3	2	80^c^
**12 mo**	53	15	3			71
**18 mo**	42	20	3		1	66

^a^ Other: CTX-M-2 (n = 7), CTX-M-32 (n = 1).

^b^ *bla*_CTX-M-1_ isolates were not tested for additional genes in the first sampling moment.

^c^ One isolate was harbouring 2 ESBL genes.

### Evaluation of interventions: Marked antimicrobial use reduction and minor changes in farm management

**Fig 2:**
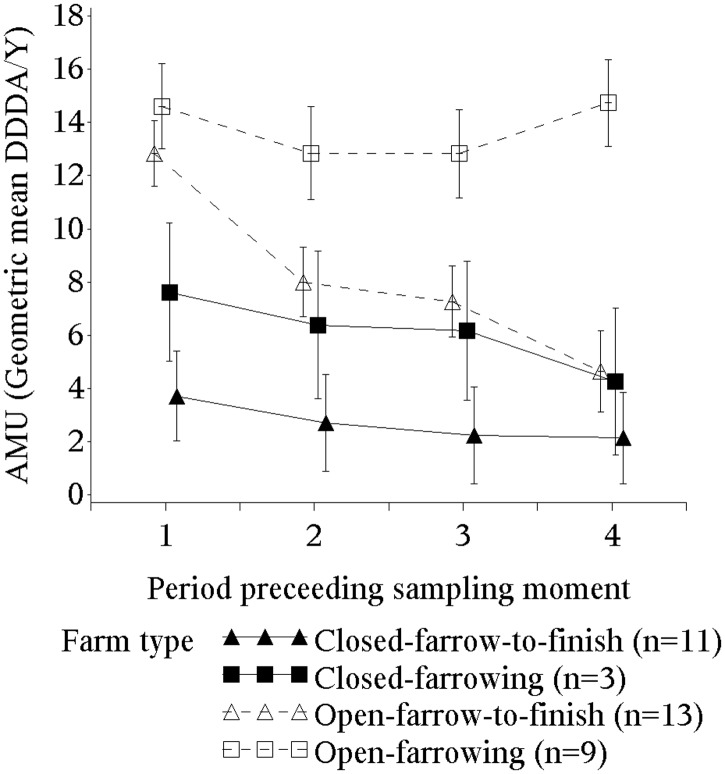
Antimicrobial use by type of farm during the 4 periods (≈6 months) before each sampling moment. GM and 95% CI from log2 DDDA/Y. AMU, antimicrobial use. Error bars indicate 95% CIs.

Farms considerably reduced AMU, likely as a result of the national benchmarking program for farms. A steady downward trend in log_2_ DDDA/Y, mirroring the overall national trend, was observed in all farm types except in farrowing open farms with a small (0.7%) increase in AMU (Fig 2). The AMU reduction was the highest in farrow-to-finish open farms (64%) and in closed farms (farrow-to-finish and farrowing) there was around a 40% reduction (Fig 2). Open farms used three times more antimicrobials as compared to closed farms (overall DDDA/Y of 9.7 and 3.1 respectively). The difference in overall AMU between open and closed farms was independent of the presence or absence of fattening pigs as shown by a non-significant interaction term between external supply and type of production. Being a farrowing farm had a multiplicative effect with a twofold increase in DDDA/Y in the strata of open and closed farms (overall DDDA/Y of 13.7, 7.7, 6.0 and 2.6 for open farrowing, open farrow-to-finish, closed farrowing and closed farrow-to-finish respectively).

During the whole study period, tetracyclines were the most used antimicrobial (37.6% of the total DDDA/Y), followed by penicillins (30.2%), trimethoprim/sulfonamides (12.3%), macrolides/lincosamides (12.0%) and polymyxins (4.6%). The last 3.3% corresponded mainly to combinations of antibiotics but also included cephalosporins, amphenicols, pleuromutilines and fluoroquinolones. Six farms used cephalosporins in the period preceding the first sampling moment, two of these farms also used these cephalosporins in the period between the first and second sampling moment. One farm only used cephalosporins in the period between the first and second sampling moment. DDDA/Y for cephalosporins varied from 0.06 to 0.39.

**Fig 3:**
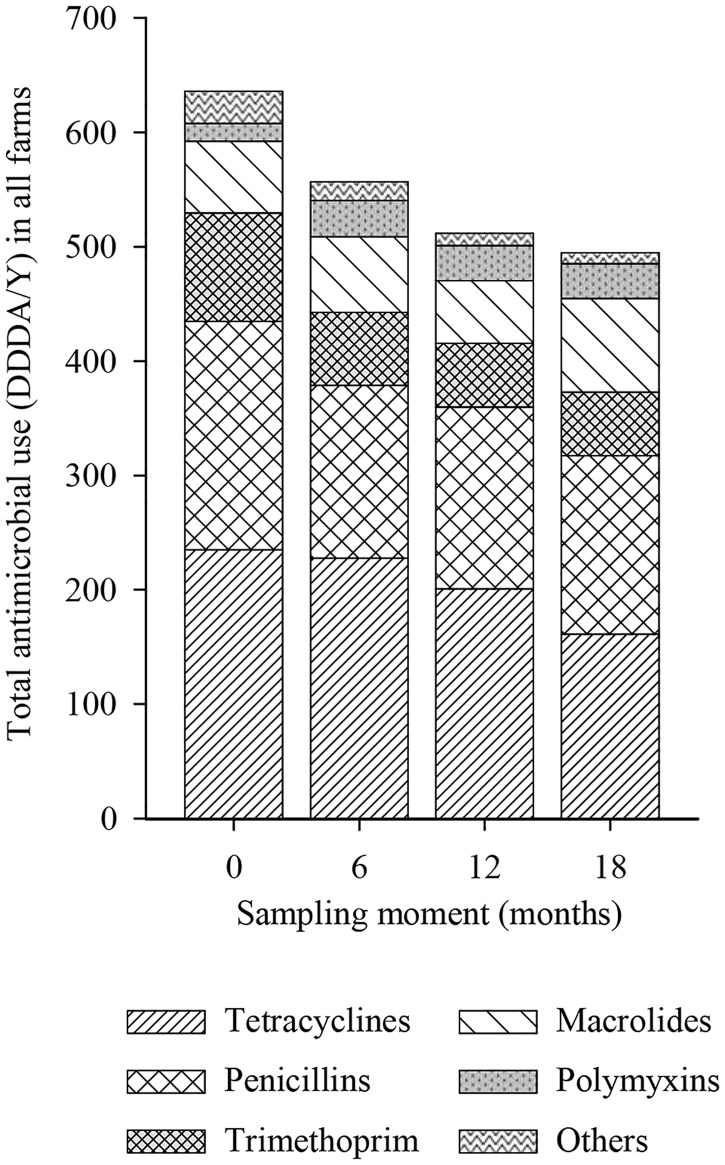
Proportions of antimicrobials used over the total DDDA/Y per farm type during the 4 periods (≈6 months) before each sampling moment. DDDA/Y, defined daily dosages animal per year.

Almost all antimicrobial classes had a parallel decrease during the study having similar DDDA/Y percentages across all the periods preceding each sampling moment (Fig 3). Only macrolides had a slight increase in percentage of DDDA/Y during the study accompanied by a slight decrease in tetracyclines and trimethoprim/sulfonamides (Fig 3). Overall, 86% of the DDDA/Y were administered as (partial) herd treatment and 13.4% as individual treatment and these percentages did not significantly differ by period of study or type of farm (not shown).

Farm management changes over time were modest; just 10% of the potential risk factors (median 9.7%, interquartile range (IQR) = 6.0–12.3) changed during the study per farm. Thus, 27 farms had changes in less than 12 variables out of the total of 134. The median number of farms within a single change was 3 (IQR = 1–4). Thus 75% of the changes occurred in four or less farms. No differences in changes over time were observed by the different farm types. Because of these limited and heterogeneous changes, an intervention effect of these changes could not be evaluated and we performed only a risk factor analysis.

### Antimicrobial use and farm management practices related to presence of *ESBL-E*. *coli* in pig farms

Univariate ORs for the presence of ESBL-*E*. *coli* carrying pigs on a farm are presented in Table 4. The probability for a farm to have ESBL-*E*. *coli* carrying pigs was 24% higher per twofold increase in DDDA/Y, but this association was not statistically significant and did not change during the course of the study (i.e. there was a non-significant interaction between sampling moment and AMU for a farm to test ESBL-positive). Stratified analysis showed this positive relation in closed farms as well, but not in open farms. Class specific DDDA/Y were not significantly associated to the presence of ESBL on pig farms. However, other variables regarding AMU were associated with ESBL-positivity of farms. When more than half of the treatments was provided to a group of pigs instead of an individual pig, the odds of a farm being ESBL-positive was approximately four times higher. The use of cephalosporins at any time in the 6 months preceding and during the study period was significantly positively associated with the presence of ESBL-*E*. *coli* carrying pigs on a farm (OR = 12.6, CI = 1.1–144.4) (Table 4).

**Table 4:** Univariate ORs for a pig farm to be ESBL-*E*. *coli*-positive.

Determinant	Category	All farms		Open farms		Closed farms	
		N^b^	OR (95% CI)	N^b^	OR (95% CI)	N^b^	OR (95% CI)
**Farm characteristics**							
No. sows	per 100 increase	144	0.69 (0.45–1.06)**	88	0.56 (0.30–1.05)**	56	0.84 (0.42–1.65)†
External supply of gilts	Open	88	6.0 (0.7–48.8)**	0	nc	56	nc
	Closed	56	Ref	88		0	
Type of production^a^	Farrow-to-finish	96	3.1 (0.4–25.4)†	52	10.3 (0.8–135.4)**	44	0.58 (0.00–72.26)†
	Farrowing	48	Ref	36	Ref	12	Ref
Water supply for animals	Public, from tap	46	0.12 (0.02–0.87)***	22	0.17 (0.01–2.61)*	24	0.15 (0.00–5.64)†
	Private source	94	Ref	63	Ref	31	Ref
Presence of goats in the farm	Yes	17	15.1 (0.8–271.8)**	10	27.2 (0.4–1863.5)*	7	28.7 (0.1–7904.0)†
	No	127	Ref	78	Ref	49	Ref
MRSA pool prevalence	per 10% increase	144	1.22 (0.94–1.58)*	88	1.18 (0.82–1.70)†	56	1.29 (0.76–2.19)†
**Biosecurity**							
Hygiene lock is the only entrance	Yes	81	0.21 (0.04–1.01)**	51	0.17 (0.02–1.22)**	30	0.17 (0.01–5.59)†
	No	62	Ref	37	Ref	25	Ref
Drivers do not enter the clean road	Yes	96	0.23 (0.05–1.18)**	50	0.21 (0.03–1.62)*	46	0.55 (0.02–18.37)†
	No	45	Ref	37	Ref	8	Ref
Dogs can enter the shed	Yes	29	5.0 (0.9–28.7)**	27	4.7 (0.7–34.0)*	2	nc
	No	115	Ref	61	Ref	54	
Removal of manure in summer	Manure stays <6 mo	123	0.21 (0.03–1.46)*	72	0.15 (0.02–1.44)**	51	nc
	Manure stays >6 mo	18	Ref	14	Ref	4	
Pest control is handed over to a professional organization	Yes	99	0.12 (0.02–0.75)***	60	0.26 (0.03–2.41)†	39	0.00 (0.00–0.26)***
	No	44	Ref	28	Ref	16	Ref
**Animal management and contact structure:**							
Foster sows can have pigs from more than one litter	Yes	75	2.5 (0.6–9.5)*	45	3.7 (0.7–19.5)*	30	0.97 (0.04–24.26)†
	No	57	Ref	34	Ref	23	Ref
Housing of gestating sows	Cubicle	69	3.3 (0.6–19.1)*	43	8.2 (1.0–68.7)***	26	0.35 (0.01–19.09)†
	Groups	69	Ref	41	Ref	28	Ref
Sick and cripple animals are taken care of in their own section^c^	Yes	29	4.7 (1.0–23.5)**	18	7.8 (1.0–59.5)***	11	0.68 (0.01–33.78)†
	No	103	Ref	59	Ref	44	Ref
Gloves always used when treating piglets	Yes	39	3.0 (0.6–15.9)*	19	4.0 (0.4–41.2)†	20	5.4 (0.2–141.9)†
	No	104	Ref	69	Ref	35	Ref
Tooth clipping in piglets	Yes	52	5.1 (0.9–29.0)**	35	5.0 (0.5–54.2)*	17	8.3 (0.2–337.6)†
	No	89	Ref	51	Ref	38	Ref
**Antimicrobial use:**							
Antimicrobial use (log2DDDA/Y)^a^ in 6 months preceding a sampling moment	per twofold increase	144	1.24 (0.84–1.84)†	88	0.88 (0.53–1.47)†	56	1.85 (0.73–4.66)*
Use of cephalosporins at any sampling moment	Yes	28	12.6 (1.1–144.4)***	24	3.92 (0.2–72.5)†	4	nc
	No	116	Ref	64	Ref	52	
Proportion of group treatments^d^	Above 0.5	100	4.0 (0.8–19.2)**	72	1.74 (0.22–13.63)†	28	7.5 (0.3–221.1)†
	Below 0.5	44	Ref	16	Ref	28	Ref

OR, odds ratio; Ref, reference category; nc, non-computable.

^a^ Items evaluated irrespective of significance.

^b^ Number of observations at all sampling times together (36 farms in 4 sampling times). Some variables have missing observations.

^c^ Variable is not selected for multivariable analysis because of having >5% of missing values over the total number of possible observations n = 144).

^d^ Variable is not selected for multivariable analysis because of high correlation with antimicrobial use (spearman rho = 0.7)

^†^ P>0.2

* p≤0.2.

** p≤0.1.

*** p≤0.05.

The presence of ESBL-*E*. *coli* carrying pigs was significantly less likely when water for the pigs was supplied from a public source instead of a private source (OR = 0.1, CI = 0.0–0.9), when a hygiene lock was the only entrance on the farm (OR = 0.2, CI = 0.0–1.0) and when pest control was carried out by a professional (OR = 0.1, CI = 0.0–0.8). There was a trend (p-value between 0.05 and 0.1) for the presence of ESBL-*E*. *coli* carrying pigs for the following determinants: external supply of gilts, presence of goats in the farm, drivers do not enter the clean road, dogs can enter the shed, sick and cripple animals are taken care of in their own section and tooth clipping in piglets (Table 4).

The results from the final model at farm and pool level are presented in Table 5. Presence of goats in the farm and the use of cephalosporins before and during the study period were risk factors for the presence of ESBL-*E*. *coli* carrying pigs on the farm (OR = 49.2, CI = 1.7->999.9 and OR = 46.4 CI = 3.1–393.1 respectively). A hygiene lock as the only entrance to the pig farm was a protective factor (OR = 0.1 CI = 0.0–0.5). The same factors were significantly associated to the presence of ESBL-*E*. *coli* in the model at the pooled pig sample level. Thereby, a significant decrease of ESBL-*E*. *coli*-positive pooled pig samples from the first to the last sampling moment was found. The presence of ESBL-*E*. *coli* was significantly different between the separate age groups in the final model at the pooled pig sample level.

**Table 5:** Multivariate ORs for a pig farm to be ESBL-*E*. *coli*-positive (Model A) and for a pooled pig sample to be ESBL-*E*. *coli*-positive (Model B).

Variable		Model A (farm level)			Model B (pooled pig sample level)		
		N	OR (95%CI)	P-value	N	OR (95%CI)	P-value
Age group	gilts	NA	NA	NA	279	0.27 (0.14–0.52)	<0.001
	finishers				183	0.48 (0.24–0.94)	
	suckling piglets				283	1.64 (0.89–3.02)	
	weaned piglets				380	0.59 (0.33–1.04)	
	sows				281	Ref	
Sampling time	0 mo	36	3.01 (0.50–18.0)	0.498	352	5.4 (2.80–10.20)	<0.001
	6 mo	36	1.11 (0.19–6.49)		356	1.78 (1.00–3.18)	
	12 mo	36	1.12 (0.19–6.50)		358	1.07 (0.60–1.90)	
	18 mo	35	Ref		340	Ref	
Presence of goats in the farm	yes	17	49.2 (1.70->999.99)	0.024	169	4.02 (1.10–15.30)	0.042
	no	126	Ref		1237	Ref	
Antimicrobial use (log2DDDA/Y)	per twofold increase	134	1.35 (0.86–2.13)	0.192	1406	0.99 (0.76–1.30)	0.943
Use of cephalosporins at any sampling moment	yes	28	46.4 (3.10–393.10)	0.006	271	72.0 (5.80–903.10)	0.001
	no	115	Ref		1135	Ref	
Hygiene lock is the only entrance	yes	81	0.06 (0.01–0.47)	0.007	797	0.06 (0.02–0.27)	<0.001
	no	62	Ref		609	Ref	

All variables in the full model were weakly correlated (spearman rho<0.4). OR, odds ratio; Ref, reference category; NA, not applicable.

## Discussion

This study suggests that the restriction in the use of cephalosporins has likely resulted in a decrease of ESBL-*E*. *coli* carriage on pig farms. ESBL-*E*. *coli* carriage in pigs significantly decreased during the study period. The observed steady reduction in total AMU did not explain these changes but the incidental use of cephalosporins was shown to be the most influential factor for ESBL-*E*. *coli* carriage of animals on farms. Additional farm management practices focused on improved biosecurity were also shown to play a role on the presence of ESBL-*E*. *coli* on pig farms.

In terms of ESBL-*E*. *coli* prevalence and gene types, other European studies have reported higher numbers of positive farms while *bla*_CTX-M-1_ gene is the most commonly found type in livestock in Europe [1,6–9].

Despite a parallel decrease of total AMU and ESBL-*E*. *coli* prevalence during the study, AMU was not significantly associated with an increased likelihood of ESBL-*E*. *coli*-positive farms. Remarkably, when cephalosporins had been applied before or during the study, the probability for a farm to have ESBL-*E*. *coli*-positive pigs was dramatically increased. Although we have to acknowledge that the confidence interval of this association was wide, its significance directly calls for the well-known causal evidence attributed to the use of these drugs for the emergence of ESBLs [2,18]. Most of the farms in this study did not use cephalosporins, which is comparable to the use in the Dutch pig farm population in the same period (2011–2013) [11,14]. We can conclude that for curbing ESBL numbers, reducing or restricting the use of cephalosporins was more decisive than an overall AMU reduction. Thereby, it can be hypothesized that the overall decrease of ESBL-*E*. *coli* carriage in pigs in this study was also a delayed result of the possible reduction in the use cephalosporins before 2011. This is in line with the fact that the farms that did use cephalosporins in this study only used it in the first two sampling moments. Cephalosporins are relatively new drugs and unlike other historically long used drugs such as tetracyclines or penicillins, resistance to cephalosporins seems not to be permanently established in bacterial communities [19]. This means that ESBL resistance might be more rapidly reverted in comparison with other resistances, as suggested elsewhere [20].

To our knowledge, evidence for risk factors for presence of ESBL-*E*. *coli* on pig farms other than AMU is very limited. A recent cross-sectional study in Germany showed that some farm management and hygienic factors could be tackled to control cefotaxime resistant *E*. *coli* [21]. In our study, the set of selected determinants in the univariate analysis showed that apart of the restricted use of cephalosporins, additional measures focused on improving biosecurity and animal management measures could be an aid to control ESBL-*E*. *coli* occurrence in pig farms. The introduction of new animals on pig farms has been reported as a risk factor for antimicrobial resistance [16,22]. In this study, a trend was seen for higher probability of ESBL-*E*. *coli* in farms with an external supply of pigs. In terms of animal age groups, the presence of ESBL-*E*. *coli* decreases over the production cycle; from suckling piglets to weaned piglets and finishing pigs, as it has been already reported by a Danish study [23]. The presence of goats in the farm as a risk factor was retained in the final model; the plausibility of this causal relationship is very doubtful and this could be just an incidental finding resulting from these farms being less strict in management and biosecurity practices (i.e. a proxy for a more poorly managed pig farm). A more specific protective factor in the multivariate model for ESBL-*E*. *coli*-positive farms was the hygiene lock as only entrance to the farm; it is quite plausible that this biosecurity measure might prevent the entrance of ESBL in the farm as suggested for other drug resistances [24]. Changes in management practices not regarding AMU were minor, therefore risk factors were probably detected more because of contrast between farms than contrast within farms over time.

We consider that results observed for our sample of farms can be generalized to the Dutch sector at large. Farms in the study contained different production types, and more importantly, their AMU was very close to national data in terms of total volumes, proportions of different antimicrobial families and proportions of individual and group animal treatments [14]. However, the differences between open and closed farms need to be cautiously interpreted since we lacked statistical power for a stratified analysis. The statistical power was also seriously compromised when a quantitative association with cephalosporins was assessed. Also, cephalosporins are only used in day-old piglets in the Netherlands. The DDDA/Y might be an underestimation because of the small total amount used due to the low weight of the piglets. Because of the limited use (in frequency and quantity) of these drugs during the study, we just evaluated their associations with ESBL-*E*. *coli* categorically. Moreover, we hypothesize that the use of cephalosporins at any point is a proxy for the cephalosporin use before 2011. Therefore excluding time variation in the use cephalosporins was justified.

Human ESBL carriage and direct contact with ESBL-*E*. *coli* carrying pigs is associated as shown by previous work [4]. This may pose a health risk for farmers and potentially for other humans with regular contact with this working population. Thereby ESBL-*E*. *coli* may be transmitted into the general population through the food chain [25]. The decreased ESBL-*E*. *coli* prevalence and the effect of cephalosporins, next to improved biosecurity and other farm management practices, showed that reduction of ESBL-*E*. *coli* on pig farms is possible. This might lead to reduced transmission of ESBL-*E*. *coli* from pigs to humans, which could be beneficial to public health.

## Supporting information

S1 Table Farm questionnaire used in each of the four sampling moments in the longitudinal risk factor analysis for ESBL-*E*. *coli* carriage in pigs. (DOCX)
