# Supplementary material for: Risk factors for ESBL-producing Escherichia coli on pig farms: A longitudinal study in the context of reduced use of antimicrobials
Source: PLoS One. 2017 Mar 21;12(3):e0174094. doi: 10.1371/journal.pone.0174094 (PMC5360262; doi:10.1371/journal.pone.0174094)
Supplement: S1 Table — (DOCX) [file pone.0174094.s001.docx]

**Supporting information**

**S1 Table. Farm questionnaire used in each of the four sampling moments in the longitudinal risk factor analysis for ESBL-*E. coli* carriage in pigs.**

| Question |  | Possible answers |
| --- | --- | --- |
| General farm characteristics:   1. Farm size: mean number of sows present per year * 2. Type of production* 3. Mean number of fattener pigs present per year 4. Frequency of pig supply per year 5. Number of farms from which pigs are supplied per year 6. External supply of gilts more than once a year from at least one supplier (aggregated from questions 3 and 4) * 7. Complete all in-all out system is applied for closed farm* 8. Frequency of removal of piglets 9. Frequency of removal of fattener pigs 10. Frequency of removal of rearing gilts 11. Frequency of removal of sows 12. Percentage of loss of weaned piglets per year 13. Average lactation period (days) 14. Mean number of weaned piglets per sow per year 15. Mean number of weaned piglets per litter 16. Percentage of loss of fattener pigs per year 17. Percentage of loss of sows per year 18. Mean growth per piglet per day 19. Mean growth per fattener pig per year 20. After delivering the leftover piglets are placed together 21. After delivering there is a leftover piglets department   Biosecurity and hygiene status   1. Hygiene status of the farm 2. The farm owns an Specific pathogen free (SPF) status* 3. SPF status for 4. Housing of the gestating sows * 5. Group size of the gestating sows* 6. There are other animals present on the farm* 7. There are also sheep present on the farm* 8. There are also goats present on the farm* 9. There are also cattle present on the farm* 10. There are also horses present on the farm* 11. There are also poultry present on the farm* 12. No other farming animals are present on the farm 13. Cats are able to enter the shed 14. Dogs are able to enter the shed* 15. Number of people working on the farm (including assisting family members) 16. Biosecurity score (aggregated sum from questions 38, 42, 45,49, 90, 136 where yes=1 and no=0 )* 17. There is only one entrance to the farm, which is the hygiene lock, other doors are locked* 18. A doorbell or phone number of the owner is clearly visible at the entrance of the farm. In this way it is possible to contact the people of the farm* 19. The farm's terrain is paved and cleaned up * 20. Silos are filled from the side of the dirty road* 21. Pigs and personnel go outside during working activities* 22. The hygiene lock consists of a clean and dirty part, separated by a passage shower* 23. The lock does not contain a shower, but it does consist of a clearly separated clean and dirty part 24. Showering is mandatory* 25. If showering is not mandatory, everyone washes his or her hands before entering the farm 26. Showering is not mandatory, however wearing farm-issued clothing is (pants and shirt 27. Farmer and his co-workers use the hygiene lock in the same way visitors do* 28. The farmer and his co-workers wash their hands before entering the farm* 29. There is warm water available* 30. There is soap available 31. There is a clean towel present 32. There are clean boots and overalls available* 33. Overalls are washed daily* 34. Overalls are washed 35. On average, for how many months does the manure stay in the pits during the summer?* 36. On average, for how many months does the manure stay in the pits during the summer?* 37. On average, for how many months does the manure stay in the pits during the winter?* 38. On average, for how many months does the manure stay in the pits during the winter?* 39. Delivered animals are placed in quarantine for a certain period of time. This part has its own entrance and is not a part of the rest of the farm 40. The quarantine has its own lock and clothing 41. The quarantine is visited at the end of the day 42. After delivery, the gilts arrive at an empty and cleaned section. This is not a quarantine 43. When gilts are delivered, these animals do not arrive at an empty section or quarantine 44. Piglets are delivered on the same day as fatteners 45. Sperm is delivered on the dirty road, the cooling box is not brought on the farm terrain* 46. There is a delivery room for materials and bagged goods. Materials are not delivered directly to the farm* 47. Pest control is handed over to a professional organization* 48. Birds are able to enter the sheds* 49. Is there presence of rats and/or mice?* 50. Animals have access to an outdoor run (e.g. after weaning) 51. When pigs are moved, they have to go outside* 52. There is a boarding platform for the sows, preventing the truck from parking directly against the shed* 53. There is a boarding platform for the piglets, preventing the truck from parking directly against the shed* 54. There is a boarding platform for the fatteners, preventing the truck from parking directly against the shed. (yes/no)* 55. There is boarding platform for piglets and/or fatteners* 56. The boarding location is not situated directly next to or beneath an air inlet* 57. The border for the delivery of animals is a 100% clear and is also implemented this way* 58. After delivery of the animals, the delivery platform is cleaned and disinfected immediately* 59. The driver does not enter the clean road* 60. Transport trucks are clean, empty and disinfected when they arrive on the farm to load the sows* 61. The carcass storage is cooled and locked* 62. The carcass cooler is situated on the dirty road* 63. Small destruction materials can be thrown into the cooler from the clean road* 64. There is a double number of barrels on the farm. So there is a surplus of barrels* 65. After the destructor emptied the barrels, the barrels are cleaned and disinfected before retrieved* 66. Rinsing water of cleaning barrels is discharged into the sewer 67. When handling carcasses, gloves are always worn* 68. When treating sick animals, gloves are always worn* 69. When treating piglets, gloves are always worn* 70. After someone entered a pen of the weaned piglets or the fatteners, hygienic measures are taken   Animal health management   1. During gestation, vaccinations are implemented 2. During lactation, vaccinations are implemented 3. The piglets and/or fatteners are vaccinated* 4. PRRSv vaccination is implemented 5. *Mycoplasma hyponeumoniae* vaccination is implemented 6. PCV2 vaccination is implemented 7. APP vaccination is implemented 8. Glässer vaccination is implemented 9. The piglets are vaccinated without the use of a needle 10. The teeth of the new-born piglets are clipped* 11. The tails of the piglets are docked* 12. The boar piglets are castrated 13. All piglets are given an injection of antibiotics in their first week of life* 14. When treating the piglets, gloves are worn 15. The gloves are renewed: 16. When treating the piglets, other hygiene measures are taken in order to prevent the transfer of infection from one to the other litter* 17. Needles for vaccination of sows are renewed:* 18. Needles for vaccination of piglets and/or fatteners are renewed per pen* 19. Needles for vaccination of piglets and/or fatteners are renewed per compartment* 20. At the end of the day, the syringes are cleaned: 21. There is a sick-bay present* 22. The sick-bay is used as a sick-bay 23. In the sick-bay, different ages are present 24. Animals enter and exit a sick-bay (back to the farm) 25. The sick-bay is visited at the end of the day 26. There is a care option for sick and cripple animals at their own group/section*   Animal contact structure   1. The sows are housed in stable groups* 2. Piglets are placed per litter* 3. Some piglets are reared motherless* 4. After the third day, piglets can still be switched* 5. Foster sows are used* 6. When creating foster sows, different litters of piglets are moved up to a different sow 7. Separation between piglet cages is open* 8. Separation between piglet cages is taken up by the feeder and/or trough, which is shared between the animals* 9. Supervision of the animals from the central hall way 10. Separation between cages fatteners is open* 11. Separation between cages fatteners is taken up by the feeder and/or trough, which is shared between the animals* 12. Separation between cages for sows is open* 13. Separation between cages for sows is taken up by the feeder and/or trough, which is shared between the animals* 14. Carcasses are placed on the ground in the section* 15. Carcasses are placed on the ground in the central hall way* 16. Cadaver bags are used* 17. Considering hygienic measures, direction of work is from young to old * 18. Sows, piglets and fatteners are different components within the farm. Each component makes use of different clothing and materials*   Cleaning and disinfection   1. All farm sections are cleaned and disinfected * 2. All farm sections are disinfected * 3. All farm sections are cleaned with soaking agents* 4. Farrow compartment hygiene (aggregated variable from questions 143 and 144)* 5. Farrow passage hygiene (aggregated variable from questions 148 and 149)* 6. Farrowing section is cleaned with cold water 7. Farrowing section is cleaned with warm water 8. Farrowing section is cleaned with soaking agent 9. Farrowing section is cleaned with disinfection agent 10. After cleaning farrowing section, there is a dry period of at least 24 hours 11. Farrowing section is cleaned by sweeping 12. Farrowing passage is cleaned with soaking agent 13. Farrowing passage is cleaned with disinfection agent 14. Piglets compartment hygiene (aggregated variable from questions 153 and 154)* 15. Piglets passage hygiene (aggregated variable from questions 157 and 158)* 16. Piglets section is cleaned with cold water 17. Piglets section is cleaned with warm water 18. Piglets section is cleaned with soaking agent 19. Piglets section is cleaned with disinfection agent 20. After cleaning piglets section, there is a dry period of at least 24 hours 21. Piglets section is cleaned by sweeping 22. Piglets passage is cleaned with soaking agent 23. Piglets passage is cleaned with disinfection agent 24. Fatteners compartment hygiene (aggregated variable from questions 163 and 164) 25. Fattener passage hygiene (aggregated variable from questions 167 and 168) 26. Fattener section is cleaned with cold water 27. Fatteners section is cleaned with warm water 28. Fatteners section is cleaned with soaking agent 29. Fatteners section is cleaned with disinfection agent 30. After cleaning fatteners section, there is a dry period of at least 24 hours 31. Fatteners section is cleaned by sweeping 32. Fatteners passage is cleaned with soaking agent 33. Fatteners passage is cleaned with disinfection agent 34. Gilts compartment hygiene (aggregated variable from questions 173 and 174)* 35. Gilts passage hygiene (aggregated variable from questions 177 and 178)* 36. Gilts section is cleaned with cold water 37. Gilts section is cleaned with warm water 38. Gilts section is cleaned with soaking agent 39. Gilts section is cleaned with disinfection agent 40. After cleaning gilts section, there is a dry period of at least 24 hours 41. Gilts section is cleaned by sweeping 42. Gilts passage is cleaned with soaking agent 43. Gilts passage is cleaned with disinfection agent 44. Mating compartment hygiene (aggregated variable from questions 183 and 184) * 45. Mating passage hygiene (aggregated variable from questions 187 and 188)* 46. Mating section is cleaned with cold water 47. Mating section is cleaned with warm water 48. Mating section is cleaned with soaking agent 49. Mating section is cleaned with disinfection agent 50. After cleaning mating section, there is a dry period of at least 24 hours 51. Mating section is cleaned by sweeping 52. Mating passage is cleaned with soaking agent 53. Mating passage is cleaned with disinfection agent 54. Gestation shed compartment hygiene (aggregated variable from questions 193 and 194)* 55. Gestation shed passage hygiene (aggregated variable from questions 197 and 198)* 56. Gestation shed is cleaned with cold water 57. Gestation shed is cleaned with warm water 58. Gestation shed is cleaned with soaking agent 59. Gestation shed is cleaned with disinfection agent 60. After cleaning gestation shed, there is a dry period of at least 24 hours 61. Gestation shed is cleaned by sweeping 62. Gestation passage is cleaned with soaking agent 63. Gestation passage is cleaned with disinfection agent   Workflow, feed and water supply   1. Work is visibly done with a week planner* 2. Work is visibly done with a day planner* 3. There are protocols present in the shed (work flows) * 4. The date of placement is present on the section doors * 5. Farm treatment plan recorded and stored* 6. A medical prescription with dosage is present on the farm* 7. Farrowing sows are fed with broth* 8. Farrowing sows are fed with dry feed* 9. Farrowing sows are fed with milk 10. Farrowing sows are fed with mush/pulp 11. Dry and gestating sows are fed with broth* 12. Dry and gestating sows are fed with dry feed* 13. Dry and gestating sows are fed with milk 14. Dry and gestating sows are fed with mush/pulp 15. Gilts are fed with broth* 16. Gilts are fed with dry feed* 17. Gilts are fed with milk 18. Gilts are fed with mush/pulp 19. Piglets with sow are fed with broth* 20. Piglets with sow are fed with dry feed* 21. Piglets with sow are fed with milk* 22. Piglets with sow are fed with mush/pulp* 23. Weaned piglets are fed with broth* 24. Weaned piglets are fed with dry feed* 25. Weaned piglets are fed with milk 26. Weaned piglets are fed with mush/pulp* 27. Fatteners are fed with broth* 28. Fatteners are fed with dry feed 29. Fatteners are fed with milk 30. Fatteners are fed with mush/pulp 31. Animals get water mainly from:* 32. Water medication is possible via a dosator* 33. Water medication is possible per section* 34. A separate medication pipe is present on the farm* 35. The water pipe is cleaned* 36. In the farrowing section drinking water is just supplied via a nipple* 37. In the farrowing section drinking water is mainly supplied via a nipple* 38. In the farrowing section drinking water is mainly supplied via a water bowl* 39. In the piglet section drinking water is just supplied via a nipple* 40. In the piglet section drinking water is mainly supplied via a nipple* 41. In the piglet section drinking water is just supplied via water bowl* 42. In the piglet section drinking water is mainly supplied via a water bowl* 43. In the fattener section drinking water is mainly supplied via a nipple* 44. In the fattener section drinking water is just supplied via water bowl* 45. In the fattener section drinking water is mainly supplied via a water bowl* 46. In the (rearing) gilt section drinking water is mainly supplied via a nipple* 47. In the (rearing) gilt section drinking water is mainly supplied via a water bowl* 48. In the mating section drinking water is mainly supplied via a nipple* 49. In the mating section drinking water is mainly supplied via a water bowl* 50. In the gestation shed drinking water is mainly supplied via a water bowl* |  | No.  Farrowing / Farrow-to-finish  No.  No.  No.  Open / Closed  Yes / No  No.  No.  No.  No.  No.  No.  No.  No.  No.  No.  No.  No.  Yes / No  Yes / No  A/ B/ C/ D/ E/ F  Yes / No  App/ Aujeszky/ M.Hyo/ PRRSv/ None  Cubicle / Groups  Yes / No  Yes / No  Yes / No  Yes / No  Yes / No  Yes / No  Yes / No  Yes / No  Yes / No  Yes / No  Yes / No  0 to 6  Yes / No  Yes / No  Yes / No  Yes / No  Yes / No  Yes / No  Yes / No  Yes / No  Yes / No  Yes / No  Yes / No  Yes / No  Yes / No  Yes / No  Yes / No  Yes / No  Yes / No  Daily/Weekly / Monthly/ Less than monthly  <6 / >6  <3 / 3-6 / 6-9 / >9  <6 / >6  <3 / 3-6 / 6-9 / >9  Yes / No  Yes / No  Yes / No  Yes / No  Yes / No  Yes / No  Yes / No  Yes / No  Yes / No  Yes / No  Yes / No  Yes / No  Yes / No  Yes / No  Yes / No  Yes / No  Yes / No  Yes / No  Yes / No  Yes / No  Yes / No  Yes / No  Yes / No  Yes / No  Yes / No  Yes / No  Yes / No  Yes / No  Yes / No  Yes / No  Yes / No  Yes / No  Yes / No  Yes / No  Yes / No  Yes / No  Yes / No  Yes / No  Yes / No  Yes / No  Yes / No  Yes / No  Yes / No  Yes / No  Yes / No  Yes / No  After each litter/ After each section /Each day  Yes / No  Once a day /  Once a week / When necessary  Yes / No  Yes / No  Daily, rinsing with cold water / Taken apart and with water and soap / Dishwasher /  Not cleaned  Yes / No  Yes / No  Yes / No  Yes / No  Yes / No  Yes / No  Yes / No  Yes / No  Yes / No  Yes / No  Yes / No  Yes / No  Yes / No  Yes / No  Yes / No  Yes / No  Yes / No  Yes / No  Yes / No  Yes / No  Yes / No  Yes / No  Yes / No  Yes / No  Yes / No  Yes / No  Yes / No  Disinfection and soaking / Just soaking / None  Disinfection and or soaking / None  Yes / No  Yes / No  Yes / No  Yes / No  Yes / No  Yes / No  Yes / No  Yes / No  Disinfection and soaking /  Just soaking /  Just disinfection / None  Disinfection and soaking / Just soaking / None  Yes / No  Yes / No  Yes / No  Yes / No  Yes / No  Yes / No  Yes / No  Yes / No  Disinfection and soaking /  Just soaking /  Just disinfection / None  Disinfection and or soaking / None  Yes / No  Yes / No  Yes / No  Yes / No  Yes / No  Yes / No  Yes / No  Yes / No  Disinfection and or soaking / Just soaking / None  Disinfection and or soaking / None  Yes / No  Yes / No  Yes / No  Yes / No  Yes / No  Yes / No  Yes / No  Yes / No  Disinfection and or soaking / None  Disinfection and or soaking / None  Yes / No  Yes / No  Yes / No  Yes / No  Yes / No  Yes / No  Yes / No  Yes / No  Disinfection and or soaking / None  Disinfection and or soaking / None  Yes / No  Yes / No  Yes / No  Yes / No  Yes / No  Yes / No  Yes / No  Yes / No  Yes / No  Yes / No  Yes / No  Yes / No  Yes / No  Yes / No  Yes / No  Yes / No  Yes / No  Yes / No  Yes / No  Yes / No  Yes / No  Yes / No  Yes / No  Yes / No  Yes / No  Yes / No  Yes / No  Yes / No  Yes / No  Yes / No  Yes / No  Yes / No  Yes / No  Yes / No  Yes / No  Yes / No  Yes / No  Yes / No  Public source, tap/ Private source  Yes / No  Yes / No  Yes / No  Yes / No  Yes / No  Yes / No  Yes / No  Yes / No  Yes / No  Yes / No  Yes / No  Yes / No  Yes / No  Yes / No  Yes / No  Yes / No  Yes / No  Yes / No  Yes / No |

* Variables with less than 10% missing values, at least 10% of farms in each category considered in the statistical analyses.
